# Supplementary figures and images for: Conservation of conformational dynamics across prokaryotic actins
Source: PLoS Comput Biol. 2019 Apr 5;15(4):e1006683. doi: 10.1371/journal.pcbi.1006683 (PMC6450608; doi:10.1371/journal.pcbi.1006683)

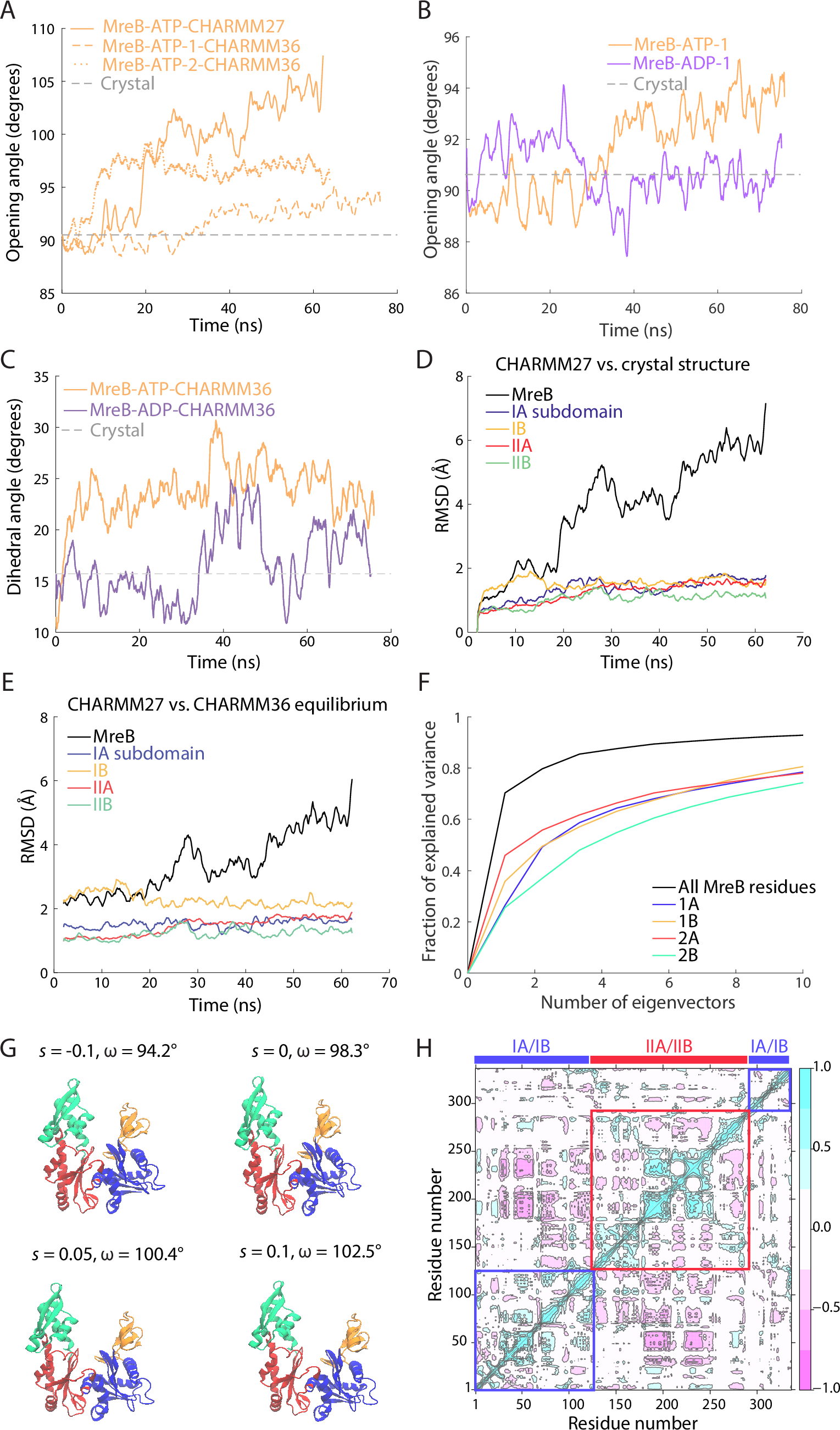

Supplement: S1 Fig — A) In CHARMM36 simulations, an ATP-bound MreB monomer adopted larger opening angles than the value in the crystal structure (dashed line). B) The trajectory of the opening angles of a MreB-ATP monomer simulation using CHARMM36 force fields shows that ATP-bound MreB stabilized with a larger opening angle than ADP-bound MreB. C) The trajectory of the dihedral angle of an MreB-ATP monomer simulation using CHARMM36 force fields shows that ATP-bound MreB stabilized with a larger dihedral angle than ADP-bound MreB. This finding is consistent with results reported using CHARMM27 force fields. D) The trajectory of the RMSD values of an MreB-ATP monomer in a CHARMM27 simulation relative to the initial equilibrated structure exhibited large changes as the protein adopted an open conformation (black line). Nonetheless, the RMSDs of the four subdomains remained ~2 Å, indicating that conformational dynamics were small within each subdomain. E) RMSD trajectories of the CHARMM27 simulation relative to the endpoint of the CHARMM36 MreB-ATP-1 simulation as the reference displayed differences in structure at the protein level. Nevertheless, the subdomains remained structurally similar. F) PCA on the trajectory of the entire protein, and separately on the trajectories of each subdomain, of an ATP-bound MreB monomer. The eigenvector with the largest eigenvalue explained 70.3% of the variance in the entire protein, versus 25.5% to 45.9% of the variance in the subdomains. The eigenvectors with the 10 largest eigenvalues collectively explained 92.2% of the variance in the entire protein, versus 78.0% to 80.6% of the variance in the subdomains. G) The locations of Cα atoms in MreB in conformations corresponding to the mean structure plus the eigenvector with the largest eigenvalue of the trajectory of an unconstrained simulation of an ATP-bound monomer, scaled by s. Conformational changes associated with movement along that eigenvector correspond to changes in the opening angle. H) Bio3D c [file pcbi.1006683.s001.tif]

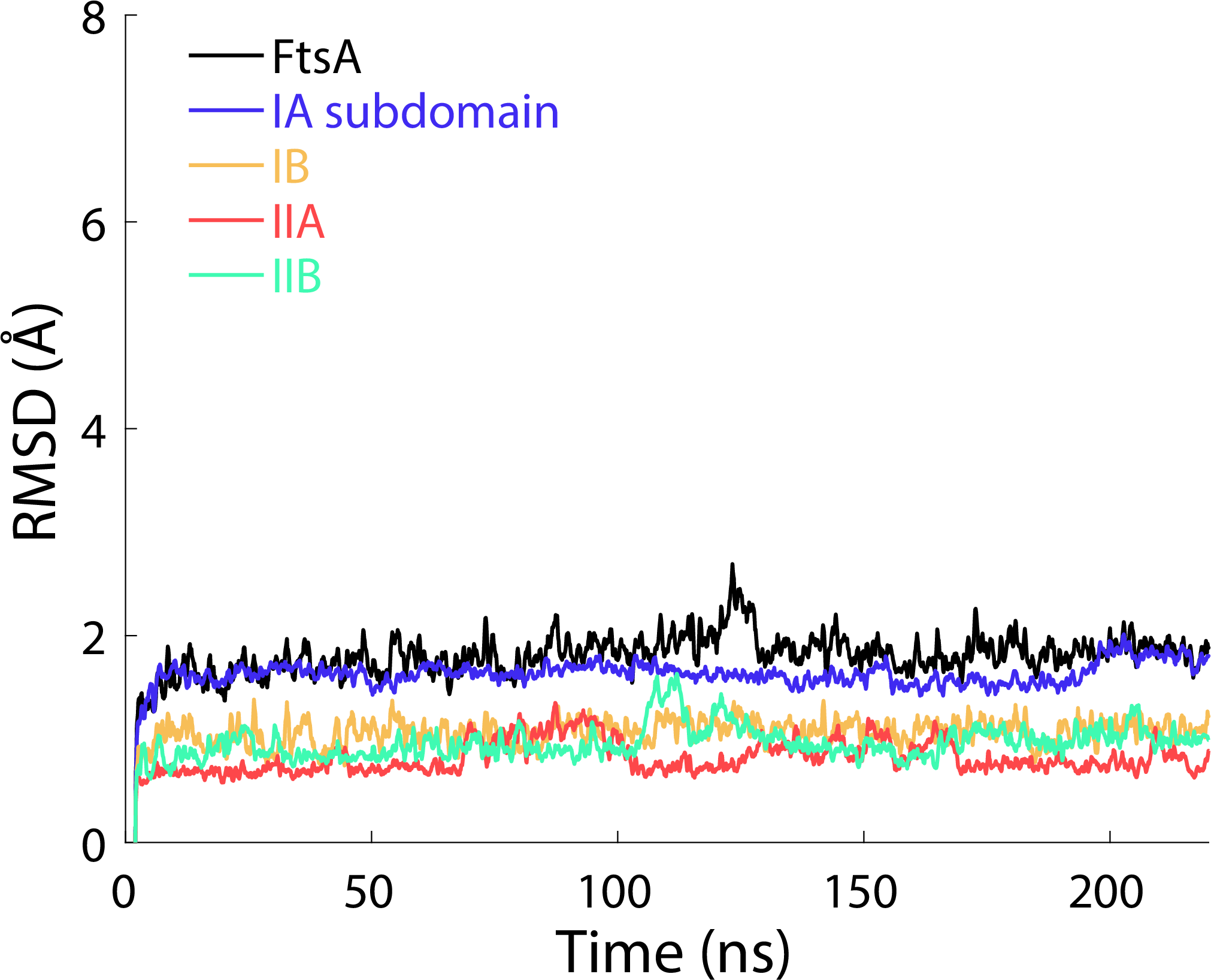

Supplement: S2 Fig — The trajectory of the RMSD values of an FtsA-ATP monomer simulation relative to the initial equilibrated structure exhibited small changes throughout the simulation (black line). Similarly, the RMSDs of the subunits of FtsA were also small. (TIF) [file pcbi.1006683.s002.tif]

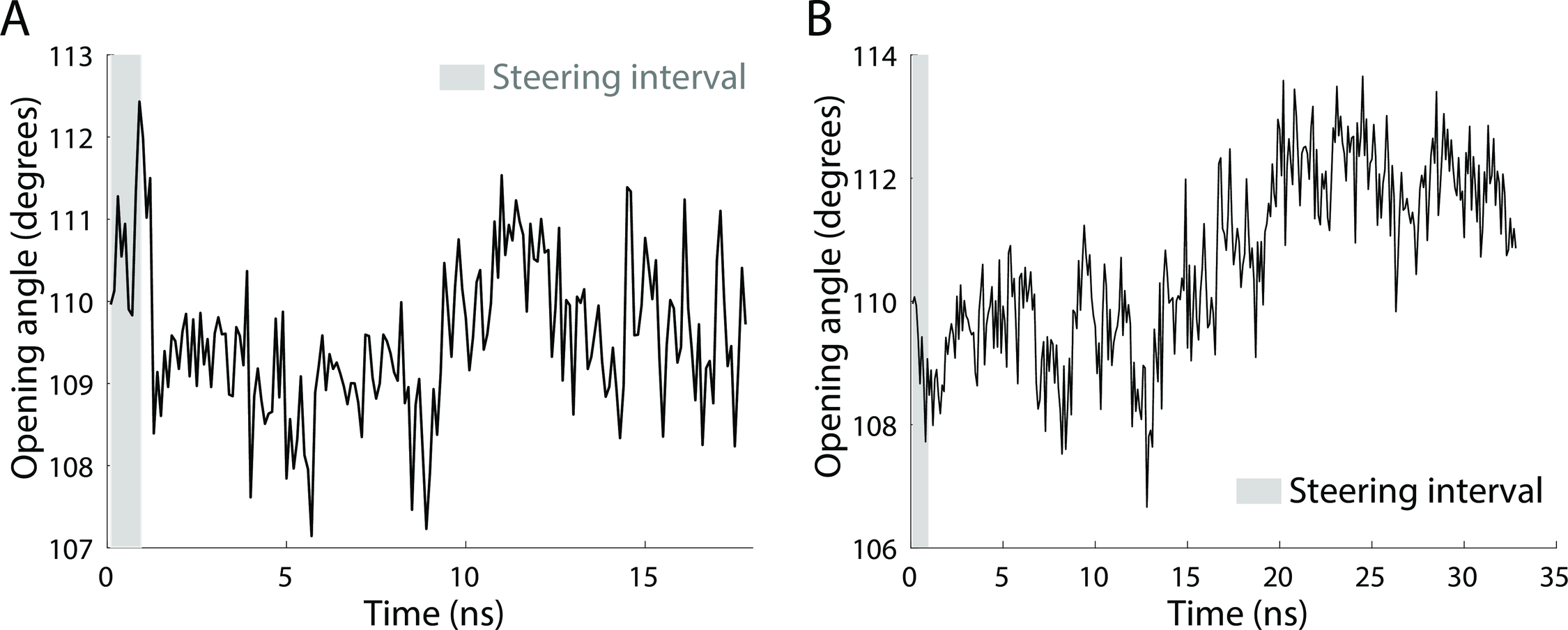

Supplement: S3 Fig — A) Trajectory of the opening angle of a steered ATP-bound FtsA monomer. The FtsA monomer was steered up to 112°. Upon release, the opening angle of the monomer stabilized at 110°, indicating that the opening angle of the ATP-bound state is stable to perturbations. B) Trajectory of the opening angle of a steered ADP-bound FtsA monomer. The FtsA monomer was steered down to 108°. Upon release, the opening angle of the monomer stabilized at 112°, indicating that the opening angle of the ADP-bound state is stable to perturbations. (TIF) [file pcbi.1006683.s003.tif]

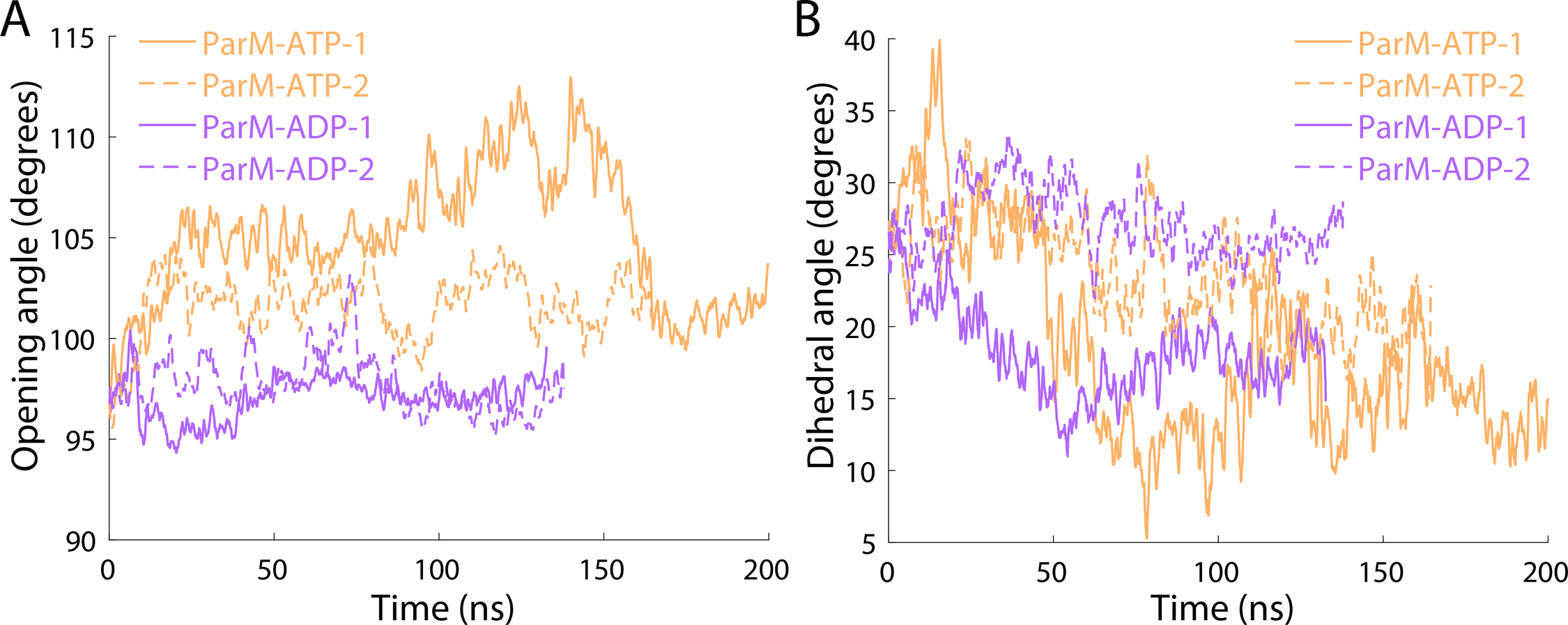

Supplement: S4 Fig — The dihedral angle (A) and opening angle (B) trajectories for two ATP-bound and two ADP-bound ParM simulations are shown. ParM dihedral angle did not display strong nucleotide dependence across replicate monomer simulations. (TIF) [file pcbi.1006683.s004.tif]

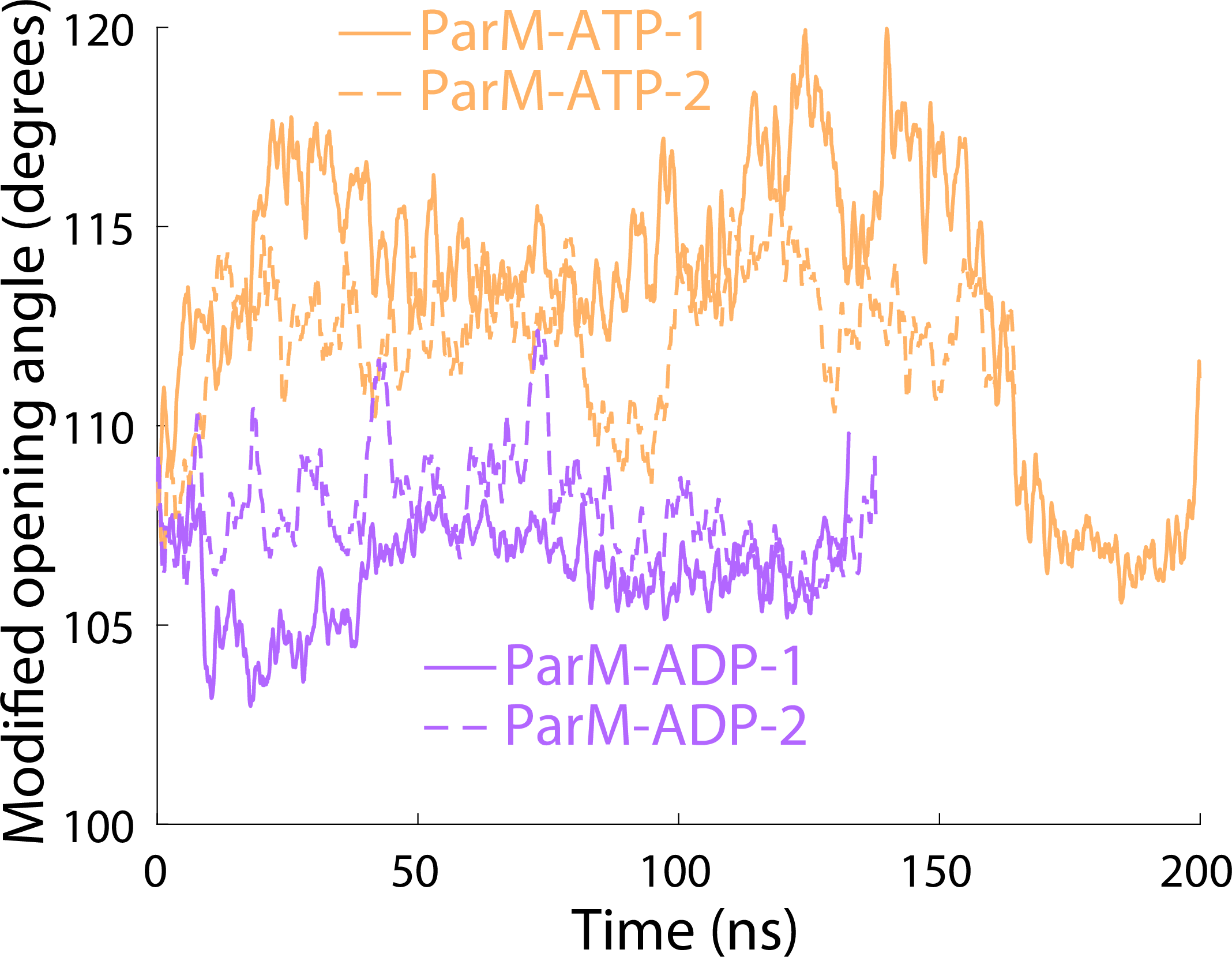

Supplement: S5 Fig — Removing high RMSF residues in ParM did not affect the nucleotide dependence of the opening angle of ParM monomers. (TIF) [file pcbi.1006683.s005.tif]

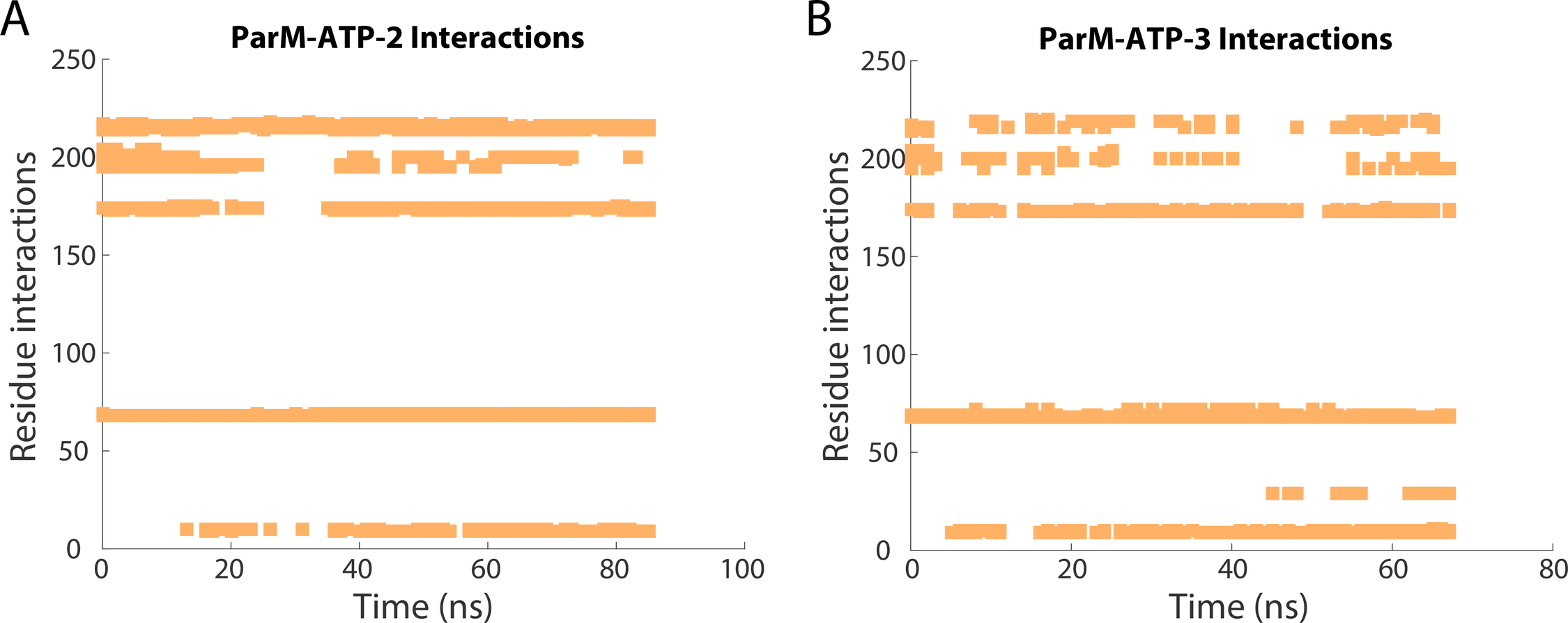

Supplement: S6 Fig — Interactions between residues 58–67 and 173–174 persisted throughout the ParM-ATP-2 (A) and ParM-ATP-3 (B) simulations, which opened much less than ParM-ATP-1, the simulation in which these interactions were disrupted (Fig 4E). (TIF) [file pcbi.1006683.s006.tif]

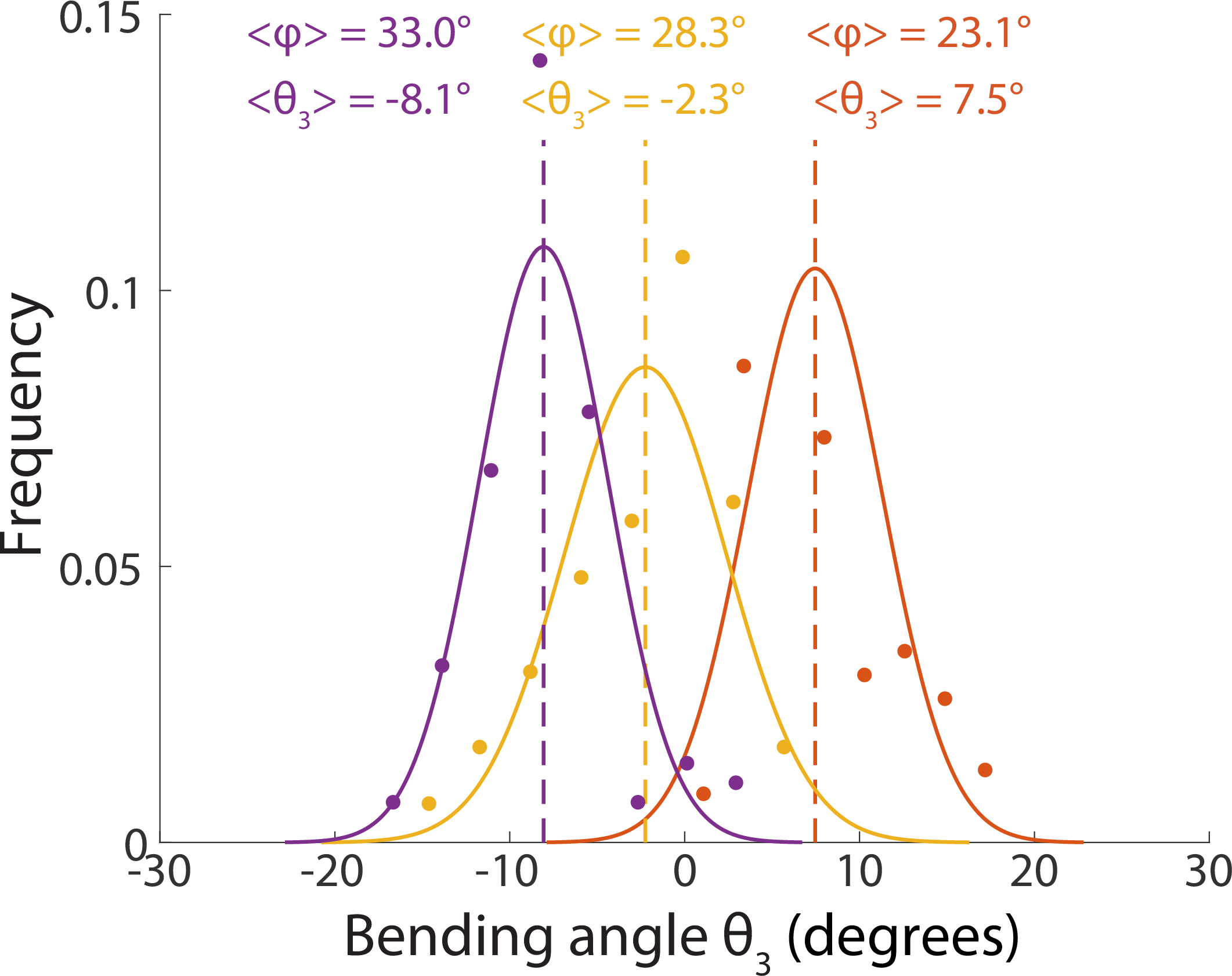

Supplement: S7 Fig — We observed an inverse relationship between the dihedral angle of the bottom subunit and filament bending in MreB dimer simulations. Dashed lines show mean values. (TIF) [file pcbi.1006683.s007.tif]

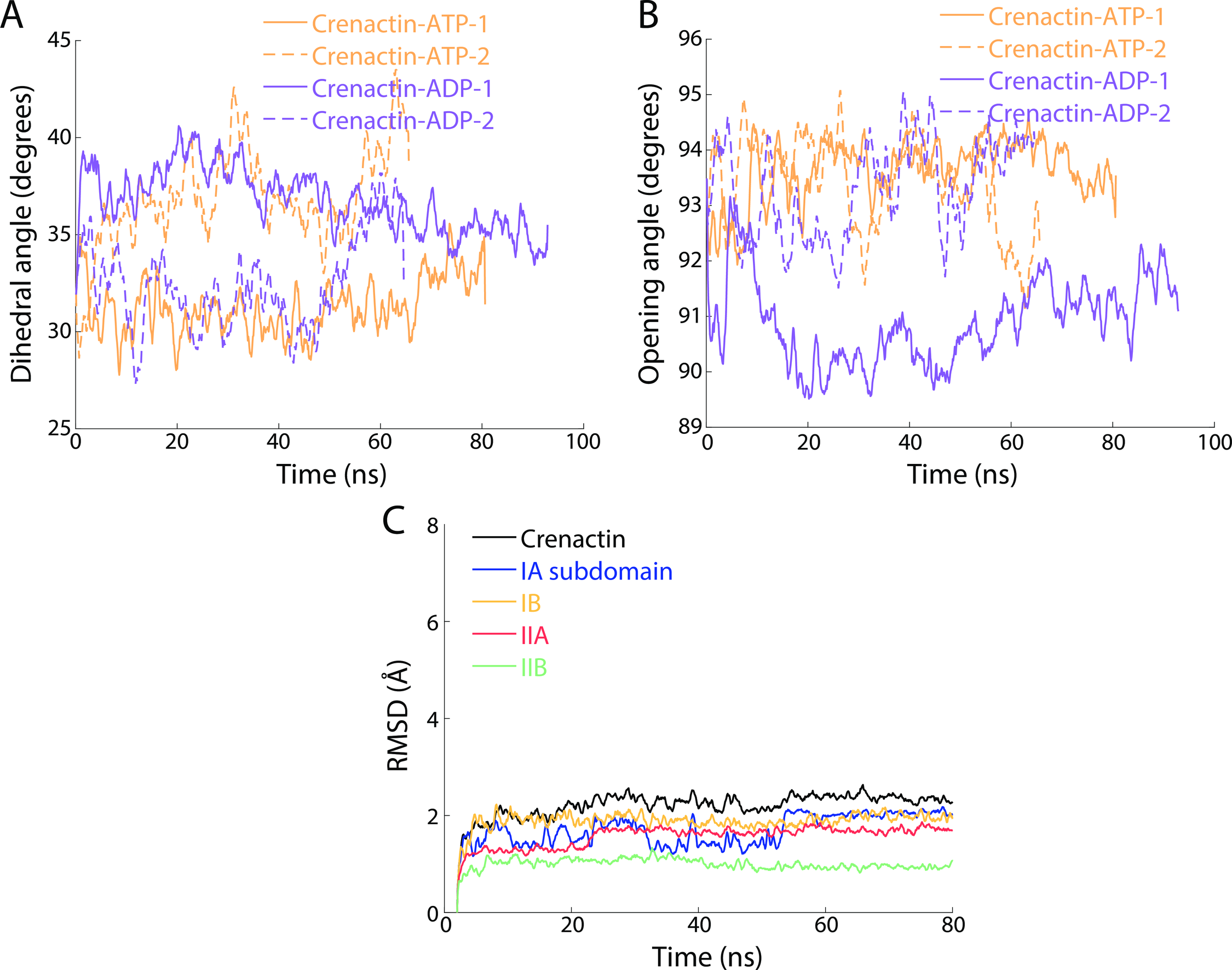

Supplement: S8 Fig — A-B) The dihedral angle (A) and opening angle (B) trajectories for two ATP-bound and two ADP-bound crenactin simulations are shown. The trajectories indicate little nucleotide dependence in monomer conformation. C) The trajectory of the RMSD values of a crenactin-ATP monomer simulation relative to the initial equilibrated structure exhibited small changes throughout the simulation (black line). Similarly, the RMSDs of the subunits of crenactin were also small. (TIF) [file pcbi.1006683.s008.tif]

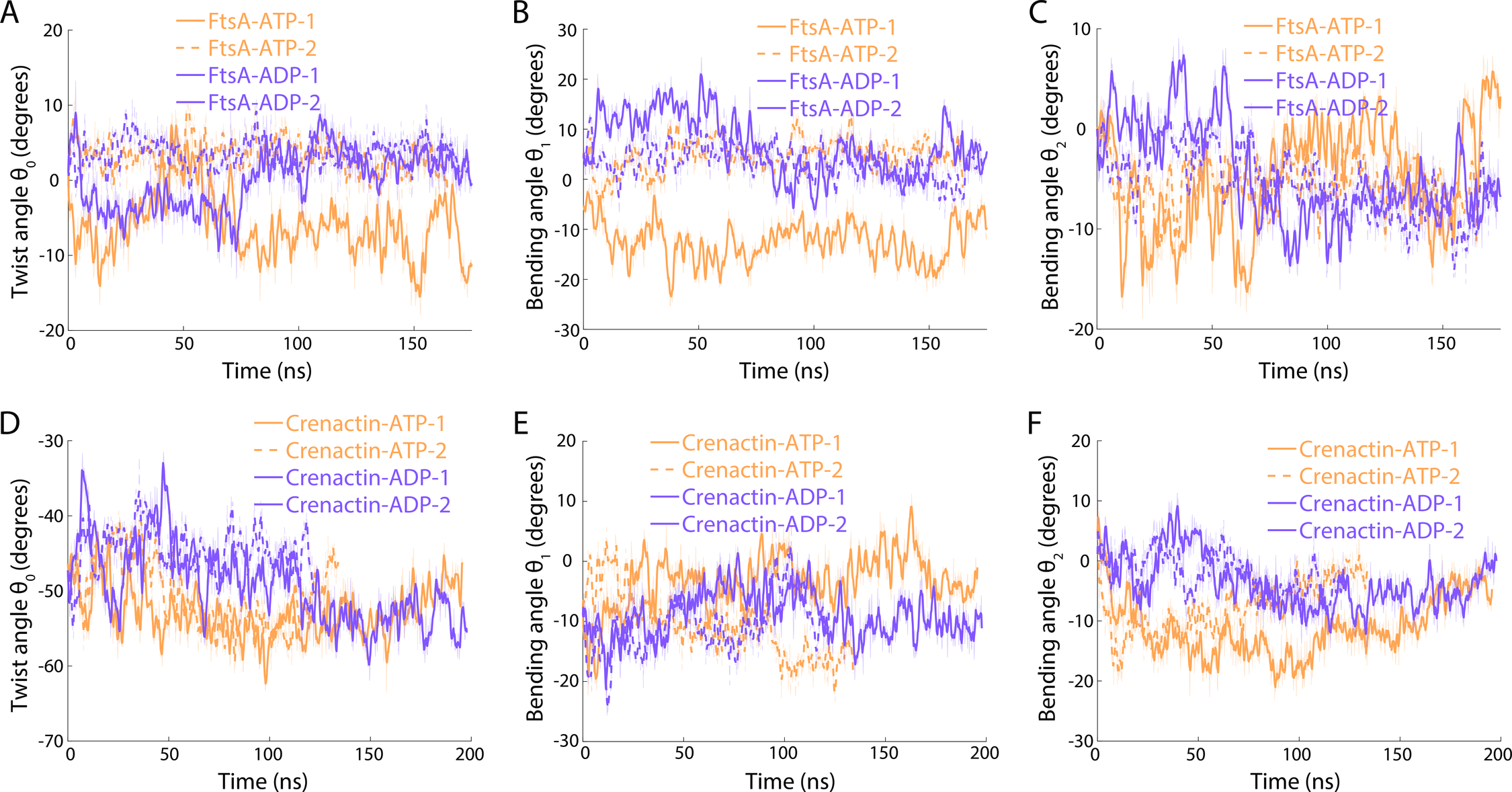

Supplement: S9 Fig — A-C) Trajectories of twisting and two bending angles for two replicate simulations each of ATP- and ADP-bound FtsA dimers. D-F) Trajectories of twisting and two bending angles for two replicate simulations each of ATP- and ADP-bound crenactin dimers. (TIF) [file pcbi.1006683.s009.tif]

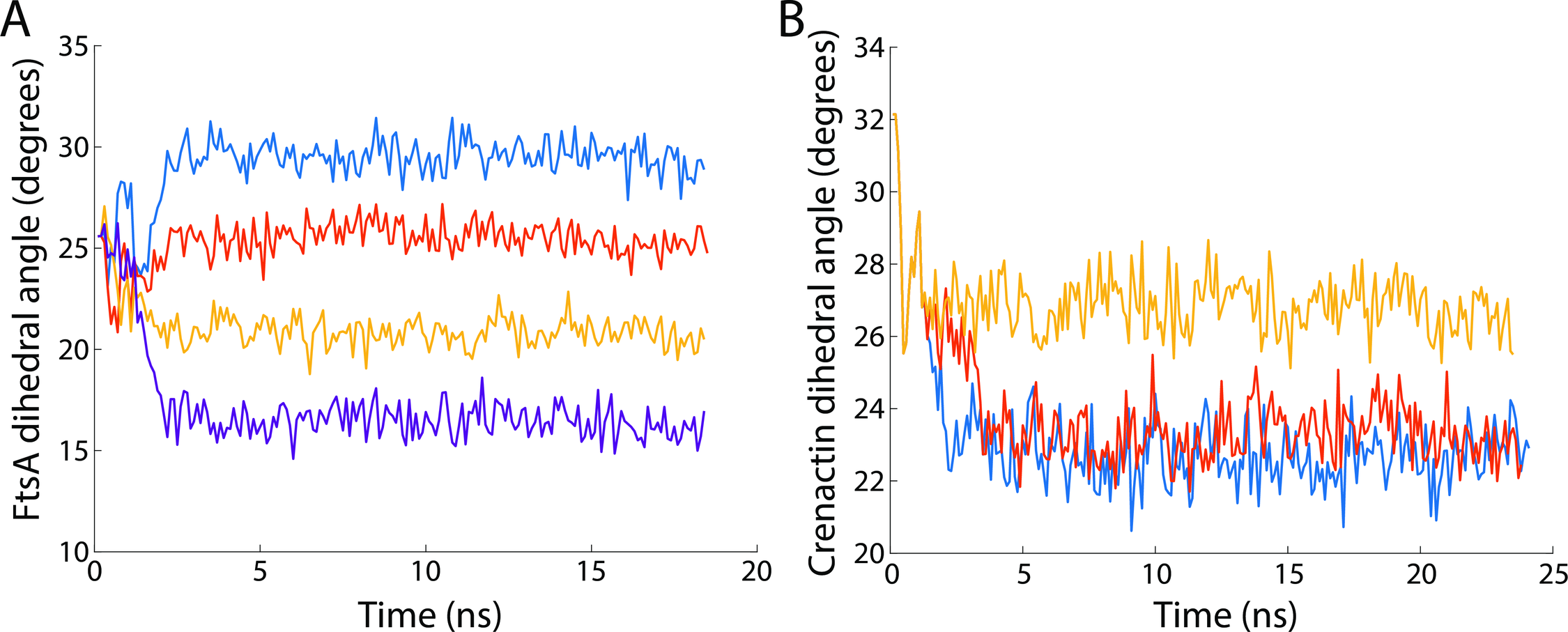

Supplement: S10 Fig — A) Trajectories of the dihedral angle of the bottom subunit in FtsA dimers in which the dihedral angle was steered to 16.4°, 20.8°, 25.5°, and 29.6°. B) Trajectories of the dihedral angle of the bottom subunit in crenactin dimers in which the dihedral angle was steered to 22.6°, 23.4°, and 26.7°. (TIF) [file pcbi.1006683.s010.tif]
