# Supplementary material for: Conservation of conformational dynamics across prokaryotic actins
Source: PLoS Comput Biol. 2019 Apr 5;15(4):e1006683. doi: 10.1371/journal.pcbi.1006683 (PMC6450608; doi:10.1371/journal.pcbi.1006683)
Supplement: S1 Table — All unconstrained simulations were replicated at least twice, with their simulation times indicated in separate rows. (DOCX) [file pcbi.1006683.s011.docx]

**Table S1****: MD simulations in this study.** All unconstrained simulations were replicated at least twice, with their simulation times indicated in separate rows.

| **Name** | **PDB structure** | **Ligand** | **Atoms (×1000)** | **Condition** | **Time (ns)** |
| --- | --- | --- | --- | --- | --- |
| 1-MreB-ATP-MG | 1JCG monomer | ATP and Mg^2+^ | 71.8 | Unconstrained | 75.9 |
|  |  |  |  |  | 63.9 |
| 1-MreB-ADP-MG | 1JCG monomer | ADP and Mg^2+^ | 71.8 | Unconstrained | 75.2 |
| 1-FtsA-ATP-MG | 4A2B monomer | ATP and Mg^2+^ | 87.2 | Unconstrained | 221.0 |
|  |  |  |  |  | 58.1 |
| 1-FtsA-ADP-MG | 4A2B monomer | ADP and Mg^2+^ | 87.2 | Unconstrained | 203.4 |
|  |  |  |  |  | 58.2 |
| 1-ParM-ATP-MG | 1MWM monomer | ATP and Mg^2+^ | 70.6 | Unconstrained | 200.0 |
|  |  |  |  |  | 164.4 |
|  |  |  |  |  | 67.4 |
| 1-ParM-ADP-MG | 1MWM monomer | ADP and Mg^2+^ | 70.6 | Unconstrained | 132.7 |
|  |  |  |  |  | 137.8 |
| 1-Crenactin-ATP-MG | 4CJ7 monomer | ATP and Mg^2+^ | 80 | Unconstrained | 80.6 |
|  |  |  |  |  | 65.6 |
| 1-Crenactin-ADP-MG | 4CJ7 monomer | ADP and Mg^2+^ | 80 | Unconstrained | 92.9 |
|  |  |  |  |  | 64.6 |
| 2-FtsA-ATP-MG | 4A2B dimer | ATP and Mg^2+^ | 116.5 | Unconstrained | 105.2 |
|  |  |  |  |  | 82.5 |
| 2-FtsA-ADP-MG | 4A2B dimer | ADP and Mg^2+^ | 116.5 | Unconstrained | 102.9 |
|  |  |  |  |  | 87 |
| 2-Crenactin-ATP-MG | 4CJ7 dimer | ATP and Mg^2+^ | 157.9 | Unconstrained | 98 |
|  |  |  |  |  | 67.2 |
| 2-Crenactin-ADP-MG | 4CJ7 dimer | ADP and Mg^2+^ | 157.9 | Unconstrained | 99.1 |
|  |  |  |  |  | 61.2 |
| 1-FtsA-ATP  *ϕ*=111.0° | 4A2B monomer | ATP and Mg^2+^ | 87.2 | Steered | 17.8 |
| 1-FtsA-ADP  *ϕ*=107.7° | 4A2B monomer | ADP and Mg^2+^ | 87.2 | Steered | 32.8 |
| 1-ParM-ATP *d*=19.3 Å | 1MWM monomer | ATP and Mg^2+^ | 70.6 | Steered | 27.3 |
| 1-ParM-ATP *d*=19.0 Å | 1MWM monomer | ATP and Mg^2+^ | 70.6 | Steered | 22.3 |
| 1-ParM-ATP *d*=14.0 Å | 1MWM monomer | ATP and Mg^2+^ | 70.6 | Steered | 30.5 |
| 1-ParM-ATP Φ=7° | 1MWM monomer | ATP and Mg^2+^ | 70.6 | Steered | 32.7 |
| 2-MreB-ATP Φ=13.0° | 1JCG dimer | ATP and Mg^2+^ | 107.8 | Steered | 11.6 |
| 2-MreB-ATP Φ=17.6° | 1JCG dimer | ATP and Mg^2+^ | 107.8 | Steered | 10.9 |
| 2-MreB-ATP Φ=22.6° | 1JCG dimer | ATP and Mg^2+^ | 107.8 | Steered | 9.2 |
| 2-MreB-ATP Φ=28.1° | 1JCG dimer | ATP and Mg^2+^ | 107.8 | Steered | 11.4 |
| 2-MreB-ATP Φ=32.5° | 1JCG dimer | ATP and Mg^2+^ | 107.8 | Steered | 10.5 |
| 2-FtsA-ATP Φ=16.3° | 4A2B dimer | ADP and Mg^2+^ | 116.5 | Steered | 18.4 |
| 2-FtsA-ATP Φ=20.8° | 4A2B dimer | ADP and Mg^2+^ | 116.5 | Steered | 18.5 |
| 2-FtsA-ATP Φ=25.3° | 4A2B dimer | ADP and Mg^2+^ | 116.5 | Steered | 18.5 |
| 2-FtsA-ATP Φ=29.5° | 4A2B dimer | ADP and Mg^2+^ | 116.5 | Steered | 18.4 |
| 2-Crenactin-ATP Φ=22.8° | 4CJ7 dimer | ADP and Mg^2+^ | 157.9 | Steered | 24.1 |
| 2-Crenactin-ATP Φ=26.7° | 4CJ7 dimer | ADP and Mg^2+^ | 157.9 | Steered | 23.5 |
| 2-Crenactin-ATP *ϕ*=31.2° | 4CJ7 dimer | ADP and Mg^2+^ | 157.9 | Steered | 24.1 |
