# Supplementary material for: Conservation of conformational dynamics across prokaryotic actins
Source: PLoS Comput Biol. 2019 Apr 5;15(4):e1006683. doi: 10.1371/journal.pcbi.1006683 (PMC6450608; doi:10.1371/journal.pcbi.1006683)
Supplement: S2 Table — (DOCX) [file pcbi.1006683.s012.docx]

**Table S2: Subdomain definitions by residue numbers.**

| **Protein** | **Structure** | **IA** | **IB** | **IIA** | **IIB** | **IC** |
| --- | --- | --- | --- | --- | --- | --- |
| MreB | 1JCG monomer | 1-29; 73-138; 315-336 | 30-72 | 139-175; 251-314 | 176-249 | N/A |
| FtsA | 4A2B monomer | 1-86; 167-198; 360-392 | N/A | 199-234; 305-359 | 235-304 | 87-166 |
| ParM | 1MWM monomer | 1-29, 68-159; 306-320 | 30-67; 130-137 | 160-198; 255-305 | 199-254 | N/A |
| Crenactin | 4CJ7 monomer | 1-38; 119-173; 399-432 | 39-118 | 174-207; 300-398 | 208-194 | N/A |
